# PELP-1 regulates adverse responses to endocrine therapy in Estrogen Receptor (ER) positive breast cancer

**DOI:** 10.18632/oncotarget.27846

**Published:** 2020-12-22

**Authors:** Michael Rees, Chris Smith, Peter Barrett-Lee, Steve Hiscox

**Affiliations:** ^1^Breast Cancer Molecular Pharmacology Group, School of Pharmacy and Pharmaceutical Sciences, Cardiff University, Cardiff, UK; ^2^Velindre Cancer Centre, Cardiff, UK

**Keywords:** breast cancer, PELP-1, ER+, invasion

## Abstract

Introduction: Endocrine therapy has played an important role in the management of ER positive breast cancer over recent decades. Despite this, not all patients respond equally to endocrine intervention, which can lead to resistance, associated disease relapse and progression. Previous reports suggest that endocrine agents themselves may induce an invasive phenotype in ER positive breast cancers with low/aberrant expression of E-cadherin. Here we investigate this phenomenon further and provide data supporting a role for the ER co-receptor, PELP-1, in mediating an adverse response to endocrine agents.

Materials and Methods: The effects of tamoxifen, fulvestrant and estrogen withdrawal (as a model for aromatase inhibitor therapy) on the invasive and migratory capacity of endocrine-sensitive MCF-7 and T47D cells, in the presence or absence of functional E-cadherin and/or PELP-1 (using siRNA knockdown), was assessed via Matrigel invasion and Boyden chamber migration assays. The effects of these endocrine therapies alongside E-cadherin/PELP-1 modulation on cell proliferation were further assessed by MTT assay. Western blotting using phospho-specific antibodies was performed to investigate signalling pathway changes associated with endocrine-induced changes in invasion and migration.

Results: Both tamoxifen and fulvestrant induced a pro-invasive and pro-migratory phenotype in ER positive breast cancer cells displaying a high basal expression of PELP-1, which was augmented in the context of poor cell-cell contact. This process occurred in a Src-dependent manner with Src inhibition reversing endocrine induced invasion/migration. While this adverse response was observed using both tamoxifen and fulvestrant therapy, it was not observed under conditions of estrogen withdrawal.

Conclusions: Our data confirms previous reports that anti-estrogens induce an adverse cell phenotype in ER+ breast cancer, particularly in the absence of homotypic cell contact. These results implicate E-cadherin and PELP-1 as potential biomarkers when deciding upon optimum adjuvant endocrine therapy, whereby tumours with high PELP-1/low E-cadherin expression may benefit from estrogen withdrawal therapy via aromatase inhibition, as opposed to ER modulation/antagonism.

## INTRODUCTION

Over the past decades, the treatment options for ER+ breast cancer have improved dramatically, with patients now likely to receive the ER modulator tamoxifen as a first line agent in the pre-menopausal setting [1], or fulvestrant as a second-line agent in locally advanced and metastatic breast cancer [2]. More recently the use of third generation aromatase inhibitors has largely replaced the use of other agents in both the adjuvant and metastatic setting, particularly in the post-menopausal age group [1]. Despite the proven efficacy of these agents, not all patients respond equally, which can lead to the acquisition of endocrine resistance and associated disease relapse or progression. Whilst adjuvant tamoxifen significantly reduces both cancer recurrence and cancer related mortality, recurrence amongst ER+ patients whilst still on tamoxifen therapy lies in the region of 25% at 10 years, with around 60% of these recurrences occurring within the first 5 years [3]. In a similar manner, patients with metastatic ER+ disease treated with fulvestrant monotherapy demonstrate an objective response to treatment in only around a third of cases, with a median time to progression of around 8 months [4], although combination treatments can be more successful [5, 6]. To date, biomarkers predictive of endocrine response outside of the ER remain scarce and better elucidation of key molecular mechanisms that predict poor response to such treatments can aid in stratification of patients for more appropriate treatments.

Src is a 60kDa non-receptor protein tyrosine kinase that has been implicated in several important oncogenic pathways [7]. As such, Src is implicated in several critical cellular processes in breast cancer, including proliferation, angiogenesis, motility and invasion [8, 9] and plays an important role in signalling cross-talk, including those mediated by the ER [10]. Src activity is increased in invasive compared with non-invasive breast cancer cell lines and invasion may be suppressed by treatment with a pharmacological Src inhibitor in these circumstances [11]. Meanwhile, activated Src expression may attenuate the response to tamoxifen and is associated with poorer survival in ER+ breast cancer patients [12]. Metastatic breast cancer patients with elevated Src expression are also associated with poorer disease specific survival [13]. Src may itself be activated by estrogen through interaction with the ER via its Src homology 2 domain (SH2), allowing further downstream signalling of MAPK and AKT, among others, through its receptor tyrosine kinase action [7]. Factors which help regulate this cascade remain unclear but may include the ER co-factor PELP-1.

PELP-1 is a large multi-domain protein which plays an important role in the modulation of several signalling cascades, including mediating the non-genomic actions of the ER [14]. Clinically, PELP-1 has been found to be an independent prognostic predictor of breast cancer-specific and disease-free survival [15] and has also been shown to be a marker associated with tamoxifen resistance, with patients whose tumours had high levels of cytoplasmic PELP1 responding poorly to treatment [16]. The protein has several known functions, such as interaction with nuclear receptors via its nuclear receptor (NR)-interacting boxes (LXXLL motifs) [17] and histone activation, through a histone binding regions located at the C-terminus [18, 19], among others. Importantly, PELP-1 contains a several PXXP motifs which facilitate interaction with proteins containing Src homology 3 (SH3) domains [14] permitting PELP-1-mediated activation of Src family kinases. Through this interaction PELP-1 can interact with several proteins that control the cell cytoskeleton, cell migration and metastases [20].

Previously we reported that a loss of the cell adhesion molecule, E-cadherin, in ER+, endocrine-sensitive breast cancer cell models resulted in an adverse, invasive response to the endocrine agents tamoxifen and fulvestrant [21]. Given the potential importance of these observations here we have explored further the cellular mechanisms that promote an adverse response to endocrine agents, and suggest a role for PELP-1 as a central mediator of this phenotype.

## RESULTS

### Tamoxifen and fulvestrant, but not E2-withdrawal, promotes invasion and migration of ER+ breast cancer cells

**Figure 1:**
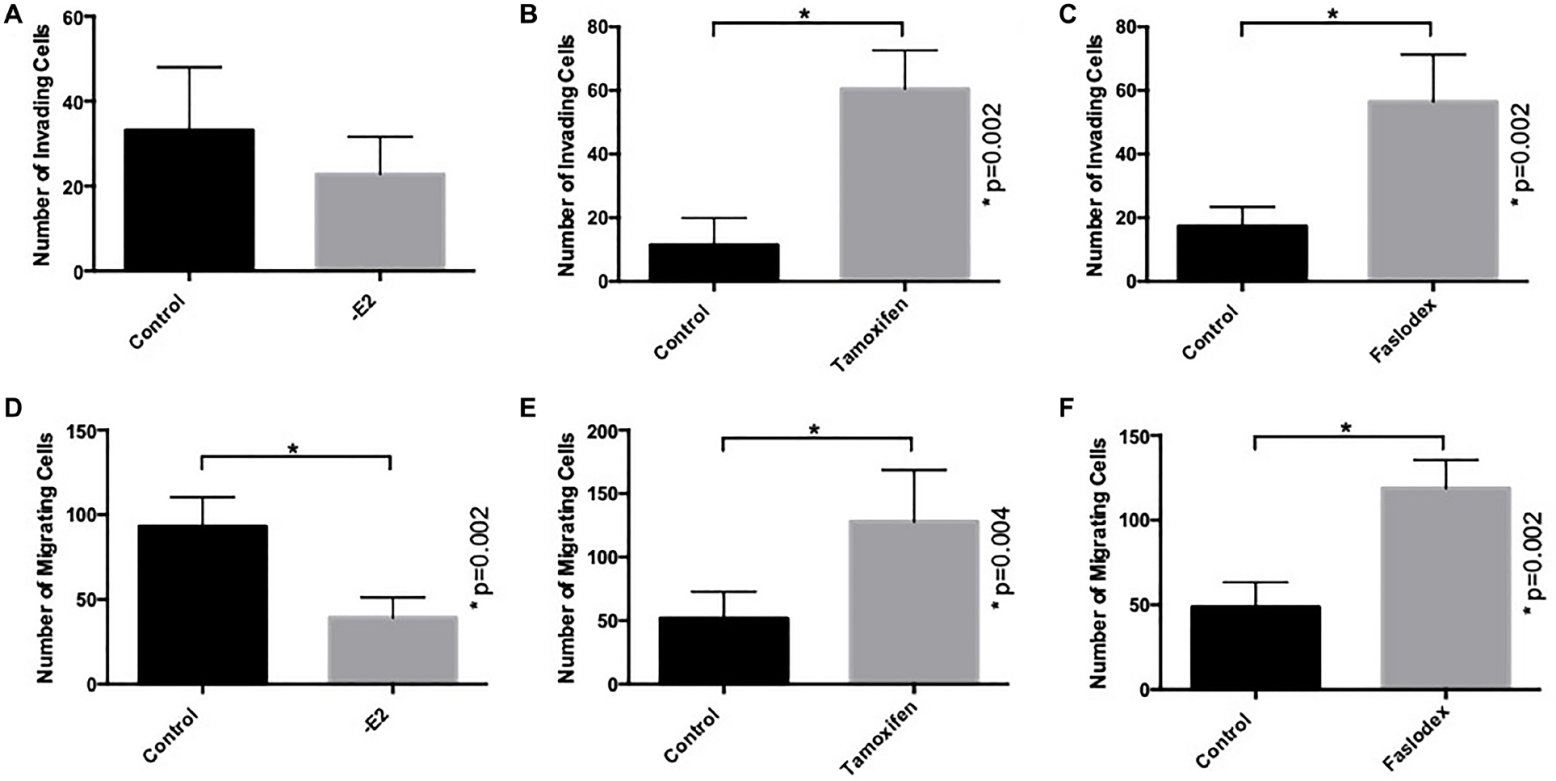
Endocrine agents induce invasion and migration of ER+ breast cancer cells *in vitro*. MCF7 cells were treated with tamoxifen, fulvestrant (both at 100 nM) or conditions of estrogen withdrawal prior to seeding into Matrigel- or fibronectin-coated Boyden chambers to measure cellular invasion (**A**–**C**) and migration (**D**–**F**) respectively.

We first examined the ability of the endocrine agents tamoxifen, fulvestrant and E2-withdrawal (as a model of aromatase inhibition) to promote the invasion and migration of ER-positive breast cancer cells as previously reported [21]. MCF-7 cells are poorly invasive *in vitro* [22] and their invasive capacity was not significantly affected by estrogen withdrawal (Figure 1A). In contrast both tamoxifen (Figure 1B) and fulvestrant treatment (Figure 1C) resulted in a significant increase in cell invasion compared to control (untreated) cells.

In a similar manner, the capacity of MCF7 cells to migrate across a fibronectin-coated, porous membrane was determined and compared to control conditions. In this case both tamoxifen (Figure 1E) and fulvestrant therapy (Figure 1F) resulted in a significant increase in the number of migratory cells whilst, in contrast, estrogen withdrawal resulted in reduced migration (Figure 1D).

### E-cadherin loss augments the pro-invasive and pro-migratory effects of endocrine agents in ER+ breast cancer cells

**Figure 2:**
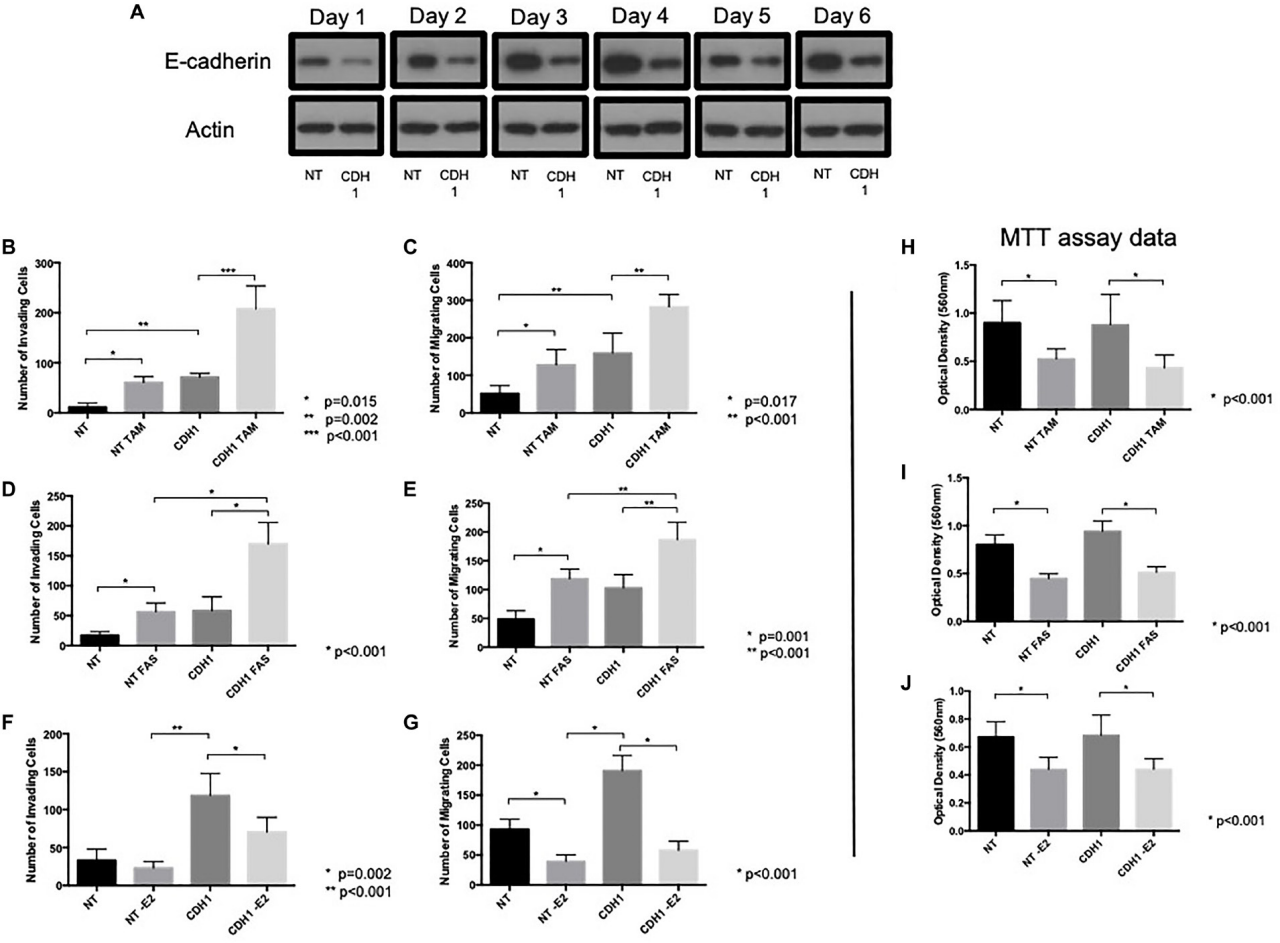
E-cadherin loss augments pro-invasive and pro-migratory actions of tamoxifen and fulvestrant. (**A**) MCF-7 cells were treated with CDH-1 siRNA and harvested 1–6 days post treatment and cellular lysates subjected to Western blot analysis to investigate whether E-cadherin expression remained suppressed following siRNA treatment. The effects of tamoxifen (**B**, **C**, **H**), fulvestrant (**D**, **E**, **I**) or estrogen withdrawal (**F**, **G**, **J**) were determined on the invasive, migratory and proliferative capacity of MCF-7 cells following siRNA-mediated E-cadherin suppression.

Previously the absence of intercellular adherens junction contacts has been suggested to enhance the pro-invasive and pro-migratory effects of tamoxifen and fulvestrant [21]. We therefore wished to investigate this phenomenon further. An siRNA approach was taken to suppress expression of the E-cadherin gene (CDH1) which resulted in a loss of E-cadherin protein, an effect that was maintained up to 6 days following exposure of cells to the agent (Figure 2A). siRNA-mediated suppression of E-cadherin resulted in a significant increase in cell invasion (Figure 2B) and migration (Figure 2C); interestingly, the ability of tamoxifen (Figure 2B and 2C) and fulvestrant (Figure 2D and 2E) to promote cell invasion and migration was significantly augmented in the absence of E-cadherin expression whereas estrogen withdrawal still did not promote an adverse cellular response (Figure 2F and 2G).

To determine whether E-cadherin knockdown altered the anti-proliferative response to endocrine agents, siRNA-treated MCF7 cells were subject to an MTT assay in the presence and absence of tamoxifen, fulvestrant and estrogen withdrawal. These data (Figure 2H–2J) confirmed MCF-7 cells were responsive to anti-hormone and estrogen withdrawal, irrespective of E-cadherin expression status and suggests that the observed changes in invasion and migration were irrespective of cellular proliferative capacity.

### Tamoxifen and fulvestrant-induced invasion and migration involves Src kinase activation

**Figure 3:**
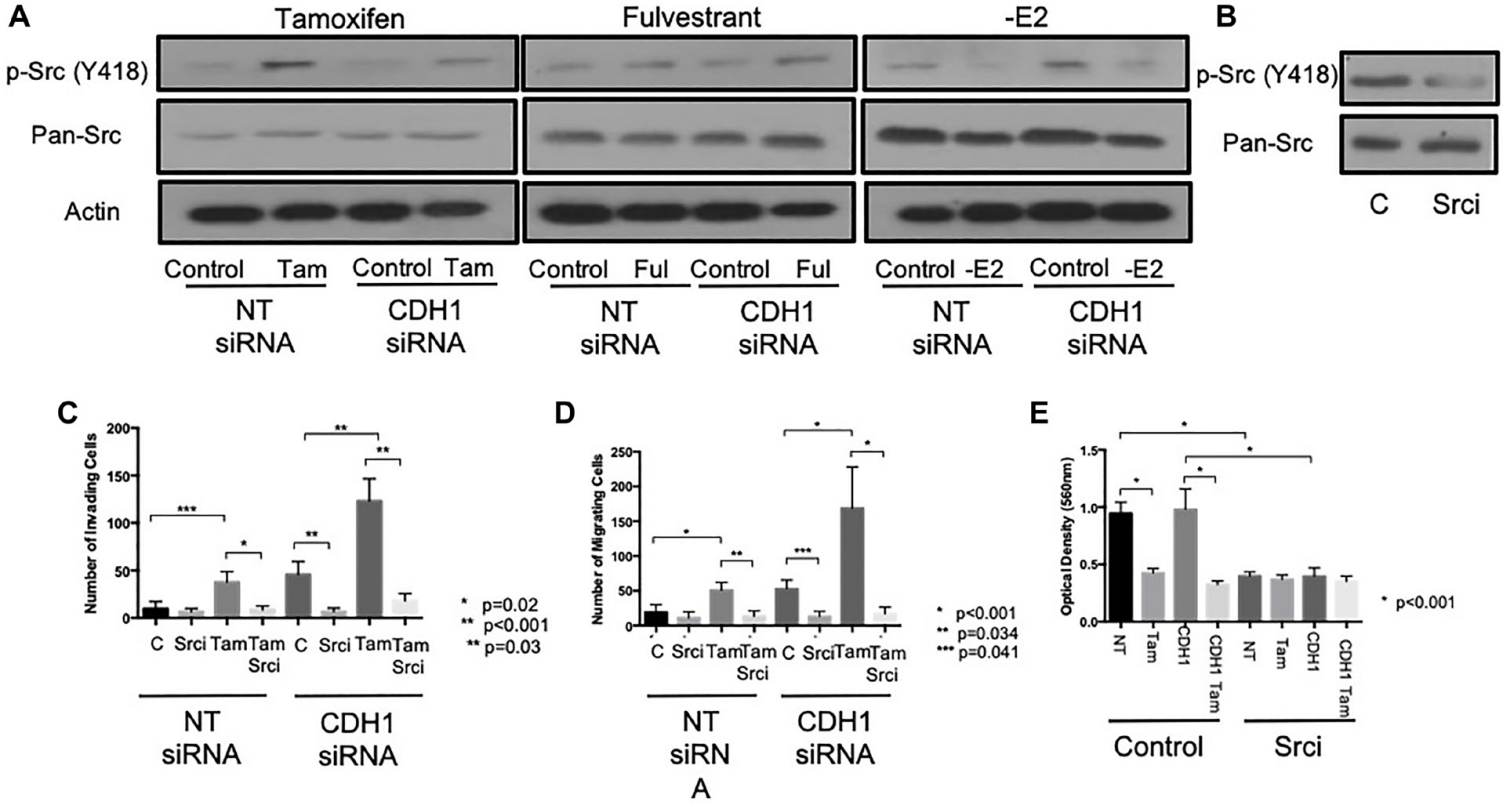
Tamoxifen and fulvestrant promote invasion of ER+ breast cancer cells through Src kinase. (**A**) The effects of tamoxifen, fulvestrant or estrogen withdrawal on Src kinase expression and activity (phosphorylation ay Y418) in E-cadherin-positive and negative MCF7 models was determined by Western blotting. The ability of the pharmacological Src inhibitor, Saracatinib (1 uM) to suppress Src activation was confirmed by Western blotting (**B**) prior to investigating the ability of Src inhibition to inhibit the pro-invasive and pro-migratory effects of tamoxifen (**C**) and fulvestrant (**D**) on MCF7 cells ± E-cadherin. Sarcatinib treatment resulted in reduce proliferation in MCF7 cells ± E-cadherin (**E**).

Tamoxifen can activate Src kinase [23] and Src is known to be a key regulator of cellular invasion and migration in acquired tamoxifen-resistant MCF-7 cells [24]. We therefore next explored whether Src kinase might play a role in the invasive behaviour observed after endocrine treatment of MCF-7 cells. In cells treated with either tamoxifen of fulvestrant, levels of phosphorylated Src (Y418) were seen to increase, an effect that occurred irrespective of E-cadherin status (Figure 3A). In contrast, estrogen withdrawal led to the suppression of Src activity in these cells.

We next sought to determine the functional relevance of Src activity in our model by performing invasion and migration assays on endocrine-treated, E-cadherin suppressed cells in the presence of the pharmacological Src inhibitor Saracatanib (1 μm). Treatment of cells with Sarcanitib resulted in the inhibition of Src kinase activity (Figure 3B) and a significant reduction in the observed invasion (Figure 3C) and migration (Figure 3D) of MCF-7 cells treated with tamoxifen ± CDH1 siRNA.

Sarcanitib was found to suppress cell proliferation in both wild type and E-cadherin - deficient cells (Figure 3E) although the combined effect of Sarcanitib and tamoxifen therapy had no additional effect on cell proliferation compared to tamoxifen alone.

### Tamoxifen and fulvestrant-induced invasion and migration, in MCF7 cells, involves an increase in ERK 1/2 and a decrease in AKT signalling

In addition to exploring Src activity and expression we further investigated whether ERK and AKT, known to be involved in Src-mediated pro-invasive signalling pathways, were altered in response to endocrine agents in the context of E-cadherin loss.

**Figure 4:**
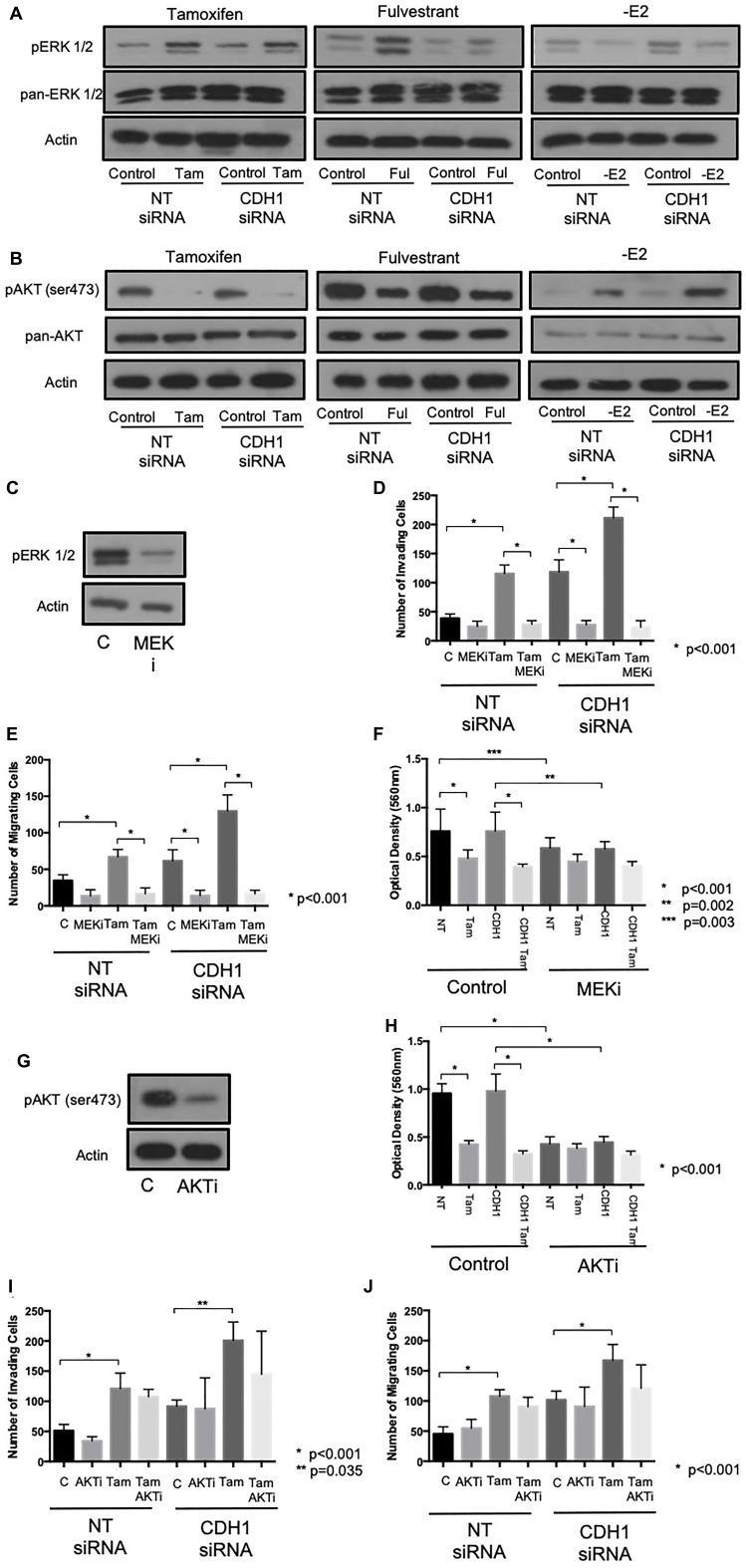
Tamoxifen and fulvestrant-mediated invasion and migration in ER+ breast cancer cells is ERK, but not AKT, dependent. The effects of tamoxifen and fulvestrant treatment on ERK1/2 (**A**) and AKT (**B**) activity in MCF7 cells ± E-cadherin expression were determined by Western blotting. The ability of the pharmacological inhibitors, U0126 (10 uM) and perifosine (10 uM) to suppress ERK1/2 and AKT activity respectively was confirmed by Western blotting (**C**, **G**) and the effects of these agents on tamoxifen-induced cellular invasion further investigated using Boyden chamber assays (U0126: **D**–**F**; perifosine: **H**–**J**).

In a similar fashion to Src kinase, phosphorylated ERK 1/2 levels were elevated in cells treated with either tamoxifen of fulvestrant, irrespective of E-cadherin status. Again, and in contrast to this, estrogen withdrawal led to the suppression of ERK 1/2 activity in these cells (Figure 4A). Phosphorylated AKT levels (Ser473) were reduced in cells treated with tamoxifen and fulvestrant, an effect that also appeared independent of E-cadherin status; estrogen withdrawal also suppressed AKT activity (Figure 4B).

The functional relevance of ERK 1/2 and AKT activity was assessed by performing invasion and migration assays on endocrine-treated, E-cadherin suppressed cells in the presence of the pharmacological inhibitors (U0126, 10 μm and Perifosine, 10 μm, respectively).

Treatment with U0126 resulted in a significant reduction in ERK 1/2 expression (Figure 4C) and a significant reduction in observed invasion (Figure 4D) and migration (Figure 4E) of MCF-7 cells treated with tamoxifen and/or CDH1 siRNA. U0126 was also found to suppress cell proliferation, assessed by MTT assay, in both wild type and E-cadherin suppressed MCF7 cells (Figure 4F). Meanwhile, while Perifosine resulted in a significant reduction of phosphorylated AKT expression (Figure 4G), and a reduction in cell proliferation (Figure 4H), AKT inhibition had no significant effect on either cell invasion (Figure 4I) or migration (Figure 4J) in these cells.

### PELP-1 expression is augmented in MCF7 cells

PELP1 is known to interact with Src family kinase SH3 domains via its PXXP motifs [14] allowing PELP1 to promote the activation of these enzymes. Given that PELP1 also binds to the ER, PELP1 may thus act to bridge Src with the ER as an alternative means of signalling, compared with direct activation of Src by the ER via the SH2 domain. Given that we have observed an invasive phenotype in response to ER modulatory agents, but not E2 withdrawal, and the fact that that this phenotype was associated with an increase in Src signalling, we wished to investigate whether PELP-1 played a role in this adverse response.

**Figure 5:**
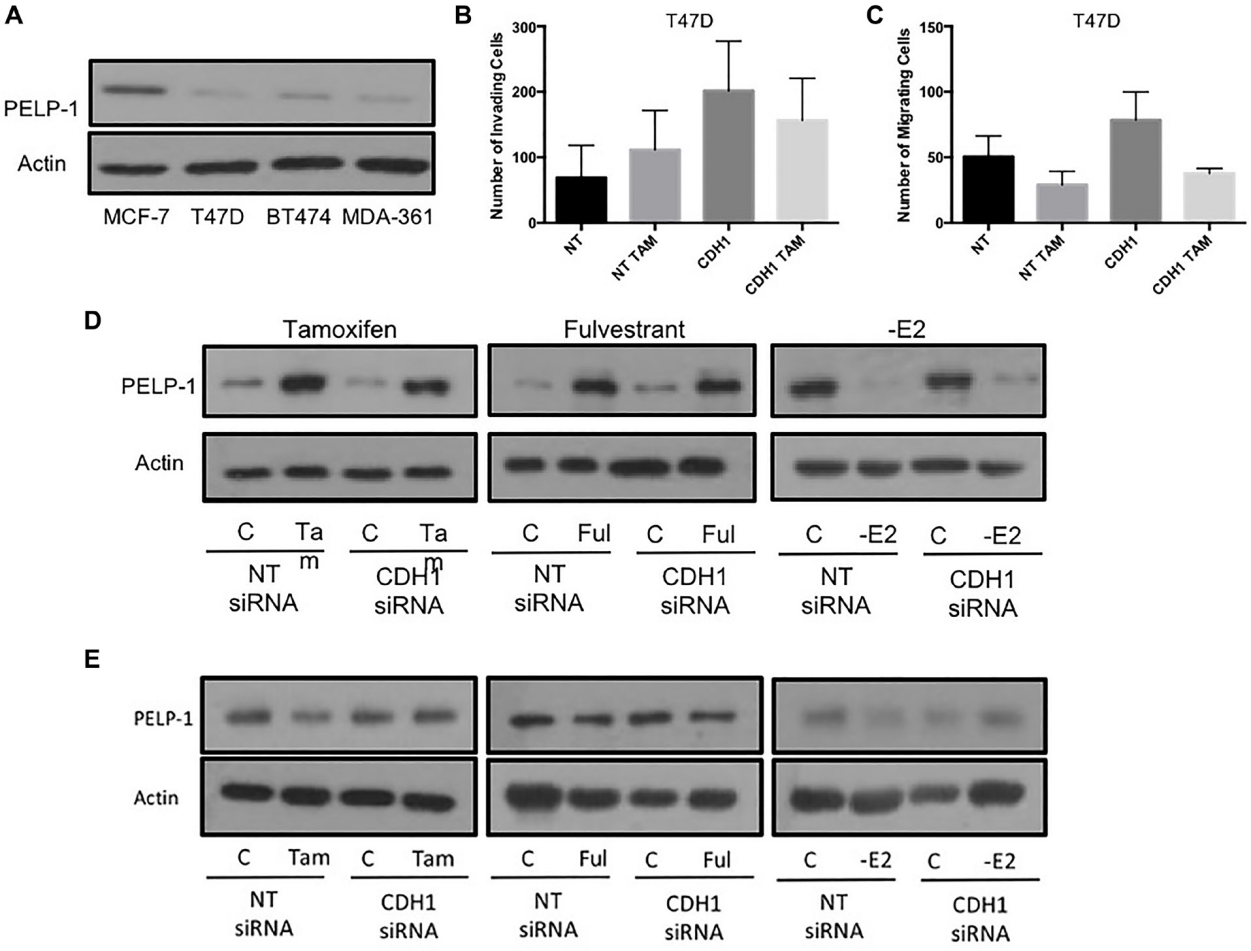
High PELP1 is associated with endocrine-induced invasion and migration in ER+ breast cancer cells. (**A**) PELP1 expression was determined in a panel of ER+ breast cancer cells using Western blotting. Subsequently, the ability of endocrine agents to induce invasion and migration in cells displaying low PELP1 expression (T47D) was investigated by Boyden chamber assays (**B** and **C**). The effects of endocrine agent on PELP1 expression were determined by examining MCF7 and T47D cell lysates using Western blotting following endocrine treatment (**D** and **E**).

Examining the levels of PELP-1 in MCF7 cells revealed that these cells had high levels of protein expression as compared to a panel of other ER+ breast cancer cell lines (Figure 5A). Using two ER+, endocrine sensitive cell models that differed intrinsically in PELP-1 expression (MCF7 and T47D), we repeated the invasion and migration assays in response to tamoxifen treatment +/– E-cadherin knockdown. In contrast to MCF7 cells (high PELP1), treatment with tamoxifen led to no significant change in invasion (Figure 5B) or migration (Figure 5C) in T47D cells (low PELP1), both in the presence and absence of E-cadherin expression. Interestingly, tamoxifen and fulvestrant both augmented the expression of PELP1 in MCF7 cells (Figure 5D), while E2 withdrawal suppressed PELP1 expression. Meanwhile in T47D cells, none of these strategies had any significant effect of PELP1 expression (Figure 5E).

### Endocrine-induced invasion and migration is abrogated in cells lacking PELP-1

**Figure 6:**
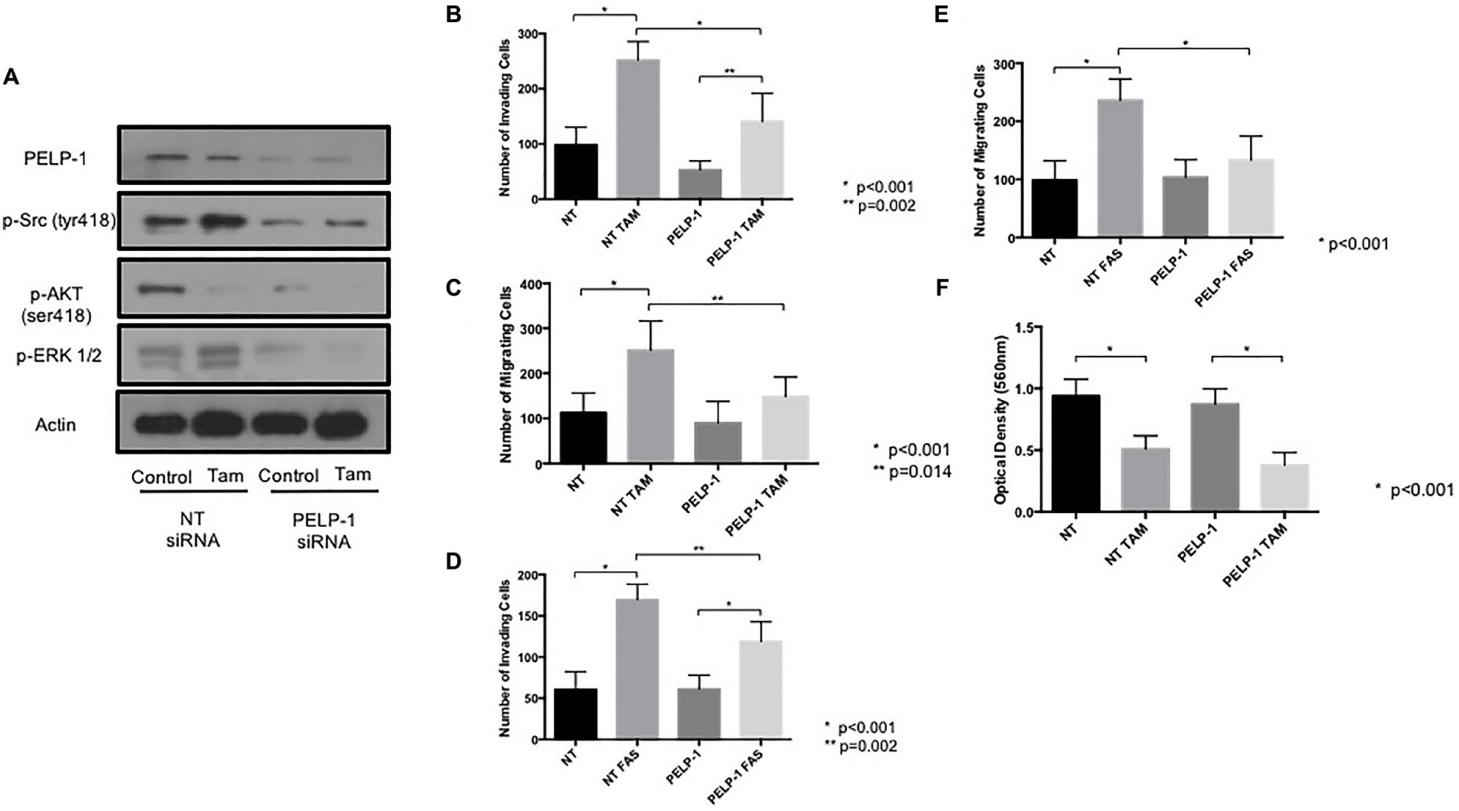
PELP1 siRNA prevents tamoxifen and fulvestrant-induced pro-invasive responses in ER+ breast cancer cells. (**A**) The effects of PELP1 modulation on internal signalling was determined by treating MCF7 cells with PELP1 siRNA and subsequently probing for PELP1, Src, AKT and ERK1/2. The effects of siRNA-mediated PELP1 suppression on endocrine-induced cellular invasion and migration were determined through Boyden chamber assays (**B**–**E**). PELP1 mediated changes in proliferation were assessed though MTT assays (**F**).

In view of this data suggesting a potential link between PELP1 and endocrine-induced invasion, we next wished to explore whether PELP-1 knockdown in MCF7 cells would affect their adverse response to endocrine agents. PELP1 expression was suppressed by siRNA prior to performing invasion and migration assays as previously. PELP-1 knockdown greatly reduced the activity of Src, AKT and ERK (Figure 6A) along with the endocrine-induced invasion (Figure 6B and 6C) and migration (Figure 6D and 6E) with both tamoxifen and fulvestrant. In contrast PELP-1 knockdown had no significant effect on cell proliferation (Figure 6F).

**Figure 7:**
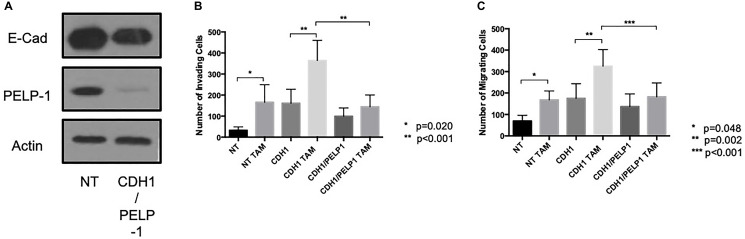
PELP 1 suppressed endocrine-induced invasion and migration in the absence of E-cadherin. Cells were treated with siRNA against PELP1 ± CDH1 (**A**) and treated with endocrine agents and the invasive and migratory capacity of these cells assessed through Boyden chamber assay (**B** and **C**).

To determine whether PELP1 also contributed to the endocrine-induced invasive responses seen to occur after E-cadherin loss, we performed a double knockdown of PELP1 and CDH1 in MCF7 cells and investigated the effects of tamoxifen and fulvestrant on these cells’ invasive and migratory nature. These data revealed that the endocrine-induced invasion and migration seen in the absence of E-cadherin was suppressed when PELP1 was removed (Figure 7A–7C)

## DISCUSSION

Adjuvant endocrine therapy has played an important role in the management of ER positive breast cancer, helping to improve overall and disease-free survival considerably over the past few decades [25]. While tamoxifen has remained the mainstay of treatment for pre-menopausal women over this time-period, more recently aromatase inhibitors have played an ever more important role in adjuvant therapy. Despite these advances, a significant proportion of women still suffer disease relapse either while still taking endocrine therapy, or after endocrine therapy has been discontinued. In addition, unwanted or adverse clinical consequences from adjuvant endocrine therapy may be under-reported or go unrecognised, leading to relapse and/or disease progression. As a result, the optimum choice of endocrine agent along with its intended duration remains unclear and it may be that as the complex effects of these agents become better understood a personalised treatment regimen may be more important depending on individual tumour biology.

Intriguingly, earlier reports suggest that endocrine agents can elicit a pro-invasive response in ER+ breast cancer cells that lack E-cadherin mediated cell-cell contacts [21]. Moreover, this paper also reported a modest increase in invasion in response to endocrine agents in E-cadherin competent cells. In our study, we have investigated these events further and confirm that an endocrine-induced adverse phenotype is apparent in ER+ breast cancer cells irrespective of E-cadherin status, albeit significantly augmented in the absence of E-cadherin. Given these observations, our hypothesis is that both tamoxifen and fulvestrant can promote a small gain in invasive and migratory behaviour, but these effects do not translate into an overly adverse cellular phenotype due to the physical constraints imposed by E-cadherin mediated cell-cell adhesion. Whilst these events were apparent upon exposure to the ER-modulatory agents, tamoxifen and fulvestrant, no increase in invasion was seen following estrogen withdrawal, as a model of aromatase inhibition.

Our investigation to explore the molecular mechanisms underlying these events highlighted a key role for Src kinase, with both tamoxifen and fulvestrant treatment resulting in an increase in Src activity, whilst estrogen withdrawal suppressed Src phosphorylation. These findings were independent of E-cadherin status. Src kinase has previously been implicated in breast cancer metastasis through its role in processes such as angiogenesis, focal adhesion and invasion, in conjunction with EMT. In terms of angiogenesis, hypoxia has been found to activate vascular endothelial growth factor (VEGF) through a Src-dependent mechanism [26, 27]. Meanwhile breast cancer cell lines have shown an increase in Src activity compared with normal breast tissue [8, 9], while conversely treatment with a pharmacological Src inhibitor results in decreased cell motility and a less invasive cellular phenotype [11]. Suppression of Src has also demonstrated reduced cell migration and attachment in MCF-7 cells through a FAK dependent mechanism [28], whilst also preventing cell rounding and detachment via its interaction with integrin [29]. Consequently, pharmacological inhibition of Src phosphorylation using Sarcanitib prevented tamoxifen and fulvestrant-induced adverse cell behaviour.

In a similar fashion to that observed when investigating Src, our results also demonstrated a role for ERK within the mechanism of adverse endocrine response. Both tamoxifen and fulvestrant therapy resulted in an increase in ERK 1/2 expression, while MEK inhibition reversed the previously observed pro-invasive/pro-migratory response of MCF-7 cells. Stimulation of the ER in MCF-7 cells has been shown to result in activation ERK in a process involving substrates of Src, which subsequently regulate the non-genomic functions of the ER in conjunction with Ras [30, 31]. Members of the MAPK family, including ERK, have been shown to be key regulators of invasion and tumour progression in breast cancer, including having roles in secretion of matrix metallo-proteinases for ECM degradation [32] and guiding cell motility [33]. In contrast to ERK, AKT function is more strongly associated with regulation of cellular growth, cell cycle progression and cell death [34]. AKT expression is also strongly associated with the development of tamoxifen resistant in breast cancer [35]. AKT is activated by a variety of stimuli through growth factor receptors such as HER2 and EGFR, in a PI3K-dependent manner [36]. From our observations tamoxifen, fulvestrant and estrogen withdrawal all resulted in suppressed activation of AKT in MCF-7 cells and while pharmacological inhibition of AKT resulted in suppressed cell proliferation, invasion and migration were unaffected.

Our data further suggests an important link between Src and the ER-coactivator, PELP-1, in the phenotypic response to endocrine agents. In addition to an increase in Src activation with both tamoxifen and fulvestrant, our data also demonstrate that both drugs appear to increase total expression of PELP-1 as compared to controls. As such, it may be possible that either PELP-1 is directly activated through an unanticipated effect of ER modulation and antagonism and/or that total levels PELP-1 levels are upregulated via a nuclear function of the ER cascade. It is also interesting to note that basal expression of PELP-1 was higher in MCF-7 cells when compared to T47D, a finding that has also previously been reported by others [17]. Tamoxifen and fulvestrant also appeared to have no effect on total levels of PELP-1 in T47D cells, in contrast to the MCF-7 cell line. It may therefore follow that the adverse response seen in MCF-7 cells may be absent from the T47D cell line because of this lower and unregulated expression of PELP-1 expression.

Importantly, whilst our data supports the role of a possible interaction between PELP-1 and Src kinase, brought about by the action of endocrine agents on the ER, further investigations are required to determine the exact nature of this interaction. Whilst PELP-1 has been shown to interact with the SH3 domain of Src via its first N terminal PxxP domain [37], its relationship based on the ligand binding status of the ER appears less clear. Given the increased expression of Src observed with both tamoxifen and fulvestrant, one would assume this change to be related to the antagonistic functions of the ER, possible resulting in ER-independent PELP1-mediated Src activation.

Although PELP-1 knockdown resulted in reduced invasion and migration in MCF-7 cells, proliferation appeared to be unaffected. This finding is despite PELP-1 knockdown resulting in lower Src kinase expression, which we have previously shown to have a significant negative effect of proliferation, in terms of pharmacological inhibition at least. This may mean that PELP-1 may be a more specific target for invasion and migration as opposed to a Src kinase itself, although it is interesting to note that as PELP-1 is a substrate of cyclin dependent kinase’s (CDK’s) [38], which regulates proliferation, while mechanistic studies have found that PELP-1 may play a permissive role in E2-mediated cell cycle progression [20, 39]. Whilst it would appear PELP-1 may be a potential future target for drug therapies, no direct inhibitors are currently available. Instead an alternative approach could be to target the downstream interactions of PELP-1, including Src, MEK/ERK and CDK2 [39], although the effects of these treatments may not have the same specificity as targeting PELP-1 itself. As a result, future drug development aiming to inhibit PELP-1 function itself, may be a potential avenue of future exploration.

Here we have demonstrated that while tamoxifen and fulvestrant resulted in an increase in invasion and migration on MCF-7 cells, estrogen suppression resulted in the opposite effect. These data again suggest that aromatase inhibition may therefore be a more appropriate treatment in tumours with low intrinsic expression of E-cadherin. While our data supports this hypothesis, it is also interesting to note that aromatase inhibition led to suppressed total levels of PELP-1 expression and reduced expression of Src kinase signalling, which further implicates PELP-1 as a potentially crucial regulator if this process. The importance of these observations have been recently investigated clinically, which further reveal that patients with ER positive cancers with low expression of E-cadherin have poorer disease free survival when treated with tamoxifen as compared to aromatase inhibitors [40].

Treatment of breast cancer is becoming more personalised with better understanding of tumour biology. Recent developments have generated tools, such as Oncotype DX^®^, which predicts the benefit of adjuvant chemotherapy based on the expression of 21 cancer-related genes [41]. Predictors of response to adjuvant endocrine therapy, outside of ER and PR expression, is currently less developed however. Identification of biological markers that could predict response to treatment may therefore prove valuable when deciding on an optimum choice of endocrine therapy. The results demonstrated in this paper would suggest that ongoing work to assess how E-cadherin and/or PELP-1 may be of value in this context would be of value.

## MATERIALS AND METHODS

### Cell culture

ER+, endocrine sensitive MCF-7 and T47D cells were routinely cultured in glutamine-supplemented RPMI medium (Invitrogen, Paisley, UK), supplemented with 5% foetal calf serum (FCS), antibiotics (10I U/ml penicillin and 10 μg/ml streptomycin) and fungi-zone (2.5 μg/ml), and incubated at 37°C with 5% carbon dioxide. For experimental analysis, the medium was changed to experimental medium, containing phenol-red-free RPMI supplemented with 5% FCS, 100 mM glutamine and antibiotics as above. For estrogen withdrawal conditions (-E2) the FCS within the experimental medium was replaced with 5% charcoal-stripped, steroid-depleted FCS, while for anti-estrogen and hormone treatments the medium was supplemented with 10^-9^ M estradiol (E2), 10^-7^ M 4-hydroxytamoxifen (‘Tam’) or 10^-7^ M fulvestrant (‘Fas’). All tissue culture media and constituents were obtained from Life Technology Europe Ltd (Paisley, UK) and tissue culture plasticware was obtained from Nunc (Rosklide, Denmark).

### Antibodies and reagents

The antibodies used were: anti-phospho Src kinase (Y418) and pan-Src kinase (Invitrogen, Paisley, UK), anti-phospho AKT (ser473), pan-AKT, anti-ERK 1/2, pan-ERK 1/2 and pan-PELP-1 (Cell Signalling Technologies, Herts, UK), anti E-cadherin antibody (R&D Systems Ltd, Oxford UK), anti-glyceraldehyde 3-phosphate dehydrogenase (GAPDH) (ABCAM, Cambridge, UK) and anti-β-Actin (Sigma-Aldrich, Poole, Dorset, UK).

### siRNA-mediated suppression of PELP-1 and E-cadherin

SMARTpool siRNA against human PELP-1 gene and human E-cadherin gene (CDH1) respectively were obtained from Dharmacon Ltd (Perbio Science UK Ltd, Northumberland, UK) and used according to the manufacturer instructions. Cells were seeded into 35 mm dishes at 10^4^ cells/dish in antibiotic-free experimental medium with or without anti-hormone as appropriate. After 24 hours of cell culture, the medium was replaced with fresh, antibiotic-free medium containing transfection lipid, 100 nM of non-targeting siRNA control (NT), 100 nM SMARTpool siRNA specific for PELP-1 or CDH1, or 50 nm SMARTpool siRNA specific for PELP-1 plus 50 nm SMARTpool siRNA specific for CDH1 where knockdown of both targets was required. Cells were assayed for PELP-1/E-cadherin protein expression after 24, 48 and 72-hours post-transfection by Western blotting to confirm protein knockdown. For invasion assays and Western blotting analysis, cells were treated with PELP-1/CDH1 siRNA for 72 hours before performing the experiments in the presence or absence of the agents as detailed.

### Basement membrane invasion assay

Cell invasion was determined using invasion chambers possessing an 8 μm porous membrane (BD Biosciences, Oxford, UK) coated with a 50 μl of 1:3 ratio or Matrigel:wRPMI. Cells (treated as above) were seeded into the top of each chamber (5 × 10^4^ cells/well) with or without anti-hormone treatment, while 650 μl of medium was added to the lower chamber of the well. Inserts were cultured at 37°C in a tissue culture incubator for 48 hours, after which the non-invasive cells and Matrigel were removed from the upper chamber of the insert with a cotton swab. The invasive cells on the underside of the insert was fixed with 3.7% formaldehyde, before the porous membrane of the insert was, detached using a scalpel blade and mounted onto a glass microscope slide using Vectashield (Molecular Probes, Eugene, OR, USA) containing the nuclear stain 4′,6-diamidino-2-phenylindole (DAPI). Cell invasion was quantified by viewing ten separate fields per membrane at a magnification of ×10 and counting the number of cells in each field. Data was then plotted as the total number of cells counted per insert +/– SD for a minimum of three independent biological replicates of the experiment.

### Cell migration assay

Cell migration was determined using invasion chambers possessing an 8 μm porous membrane (BD Biosciences, Oxford, UK) coated with an air-dried solution of fibronectin mixed with RPMI (1:100). Cells (treated as above) were seeded into the top of each chamber (4 × 10^4^ cells/well) with or without anti-hormone treatment, while 650 μl of medium was added to the lower chamber of the well. Inserts were cultured at 37°C in a tissue culture incubator for 24 hours, after which the non-invasive cells and Matrigel were removed from the upper chamber of the insert with a cotton swab. The migratory cells on the underside of the insert were fixed with 3.7% formaldehyde, before the insert was stained with crystal violet solution. Cell migration was quantified by viewing ten separate fields per membrane at a magnification of ×10 and counting the number of cells in each field. Data was then plotted as the total number of cells counted per insert +/– SD for a minimum of three independent biological replicates of the experiment.

### Cell lysis and western blotting

After cell culture were treated as described above, cells were washed twice with ice-cold PBS and lysed in lysis buffer (50 mM Tris, pH 7.5, 5 mM EGTA, 150 mM NaCl, 1% Trixton X100) containing protease inhibitors (2 mM sodium orthovanadate, 20 mM sodium fluoride, 1 mM phenyl-methylsulfonyl fluoride, 20 μM phenylarsinine, 10 μM sodium molybdate, 10 μg/ml leupeptin and 8 μg/ml aprotinin). The lysates were then placed on ice for 20 minutes and clarified by centrifugation (15 minutes, 15,000 rpm, 4°C). The concentration of solubilised proteins was then determined using the DC protein assay kit (BioRad, Hemel Hempstead, UK). Using these lysates, 20 μg of total protein was separated by SDS-PAGE using 10% gels and transferred to nitrocellulose membranes by electroblotting. Membranes were then blocked using 5% (w/v) milk protein in Tris-buffered saline containing 0.05% Tween-20 and incubated with primary, followed by secondary horseradish peroxidase-conjugated secondary antibodies. An enhanced chemiluminescence system (West ECL reagent, Pierce and Warriner Ltd, Chester, UK) was used for detection of bound antibodies by exposing the blots to X-ray film (Kodak, UK). Blots shown are representative of a minimum of three separate biological replicates of the experiment.

### Cell proliferation assay(s)

For the MTT assay, cells were seeded into a 96-well plate (1 × 10^6^cells/plate) and, after 24 hours, appropriate treatment were added, as described, and cells cultured for a further 72 hours. For analysis of the effects of PLEP-1/CDH1 knockdown on basal growth rates, cells were pre-treated with siRNA, seeded into the plates and cultured for 72 hours with no further treatment. Cells were then washed gently with warm phosphate buffered saline (PBS) and incubated with 3-(4,5-dimethylthiazol-2-yl)-2,5-diphenyl-2H-tetrazolium dehydrogenase (MTT) at 37°C for 4 hours to allow formation of formazan crystals within mitochondria. The MTT was then replaced with Trixton-X-100 (Sigma-Aldrich, Poole, Dorset, UK) and the plate maintained at 4°C overnight, to allow the formazan crystals produced to be released from mitochondria and dissolve. Data was obtained by recording the optical density of each well using an ELISA plate reader (mean of eight separate wells per condition), with experiments repeated using a minimum of three biological replicates.

### Statistical analysis

All graphical data is presented using the Prism Graphpad^®^ version 6 statistical software. Statistical analysis was performed using SPSS^®^ 20. Statistical significance was determined by a *p*-value of < 0.05. For analysis of data comparing two independent variables an independent samples *t*-test was performed where data followed a normal distribution, while a Mann-Whitney test was performed in cases where the data was deemed to be non-parametric. For comparison of multiple variables, a one-way ANOVA test was performed to assess for a significance across the dataset, with a post-hoc Bonfferoni test used to assess significance between two of the variables within the dataset.
